# Bioinspired Networks of Communicating Synthetic Protocells

**DOI:** 10.3389/fmolb.2021.804717

**Published:** 2021-12-24

**Authors:** Patrick J. Grimes, Agostino Galanti, Pierangelo Gobbo

**Affiliations:** ^1^ School of Chemistry, University of Bristol, Cantock’s Close, Bristol, United Kingdom; ^2^ Department of Chemical and Pharmaceutical Sciences, University of Trieste, Trieste, Italy

**Keywords:** bottom-up synthetic biology, protocell, chemical communication, prototissue, protocellular material, out-of-equilibrium system, intelligent material, active matter

## Abstract

The bottom-up synthesis of cell-like entities or *protocells* from inanimate molecules and materials is one of the grand challenges of our time. In the past decade, researchers in the emerging field of *bottom-up synthetic biology* have developed different protocell models and engineered them to mimic one or more abilities of biological cells, such as information transcription and translation, adhesion, and enzyme-mediated metabolism. Whilst thus far efforts have focused on increasing the biochemical complexity of individual protocells, an emerging challenge in bottom-up synthetic biology is the development of networks of communicating synthetic protocells. The possibility of engineering multi-protocellular systems capable of sending and receiving chemical signals to trigger individual or collective programmed cell-like behaviours or for communicating with living cells and tissues would lead to major scientific breakthroughs with important applications in biotechnology, tissue engineering and regenerative medicine. This mini-review will discuss this new, emerging area of bottom-up synthetic biology and will introduce three types of bioinspired networks of communicating synthetic protocells that have recently emerged.

## Introduction

Communication between cells *via* diffusible chemical signals is one of the essential pillars of life. From bacteria to higher mammals, cells exchange information between themselves to reproduce, differentiate, coordinate metabolism and gene expression, regulate population density, direct migration and assemble into complex and macroscopic three-dimensional (3D) structures. In order to test important modern hypotheses of cell biology and to better understand the mechanisms and systems utilised by living cells to communicate, researchers working in the field of synthetic biology have attempted to synthesize various cell mimics from inanimate building blocks following an *understanding-by-building* approach. This novel methodology is known as *bottom-up* synthetic biology, meaning that existing biological systems are not modified using synthetic biology methods, but rather wholly abiotic molecules and materials are assembled in ways that mimic and emulate fundamental living structures, such as cells (Luisi et al., 2006; Altamura et al., 2021a). The result of this body of research, which is based on an assortment of fundamental early works (Szostak et al., 2001; Luisi, 2002; Pohorille and Deamer, 2002; Noireaux and Libchaber, 2004; Swi Chang, 2007), is a broad array of different cellular mimics, ranging from liposomes (Rideau et al., 2018) and lipid vesicles (Vieregg and Tang, 2016) to polymersomes (Palivan et al., 2016; Che, Buddingh’, van Hest), dendrimerosomes (Jiang et al., 2015), coacervates (Koga et al., 2011; Mason et al., 2017), proteinosomes (Huang et al., 2013; Huang et al., 2014a; Huang et al., 2014b), as well as models based on synthetic peptide or nucleic acid membranes (Vogele et al., 2018; Huber et al., 2019; Schreiber et al., 2019; Samanta et al., 2020). Although these represent the most popular protocell models developed so far, a complete list with advantages and disadvantages has been the subject of several comprehensive reviews (Rideau et al., 2018; Kamiya, 2020; Martin and Douliez, 2021; Buddingh’ and van Hest, 2017; Li et al., 2014). From a general perspective, this array of protocell models can be divided into two broad categories: typical and non-typical (Xu et al., 2016). *Typical* protocells are those which demonstrate a key feature of living cells, such as DNA transcription/translation, the ability to grow and divide or the metabolism of molecules to generate energy for the protocells’ use. Conversely, *non-typical* protocells are much less strict in their criteria for construction and are not limited in terms of their building materials or their direct similarity to biological cells. Non-typical protocells have several advantages in application as greater scope exists to combine them with harsh/inhospitable conditions or with reagents which may be toxic to living cells. Moreover, there is greater freedom to synthesise entirely new, non-equilibrium micro-compartmentalised chemical systems that can coexist in parallel to well-known biological ones.

Whilst the focus in this research field for the past decade has been on the development and characterisation of protocell models, a novel area that has recently emerged in a transversal way and is increasingly attracting attention is the design and synthetic construction of networks of communicating synthetic protocells. Inter-protocellular communication can be defined as the exchange of chemical signals between individual protocells, or discrete populations of protocells, which initiates some form of chemical response in the receivers of said signals. There has been rapid progress in this new area of protocell engineering and the disparate examples reported in the literature have undergone some categorisation in existing reviews (Rampioni et al., 2019; Luan et al., 2020; de Luis et al., 2021; Mukwaya et al., 2021). However, these examples have not, as yet, been collated into a concise discussion of the methods, systems and strategies used to synthetically mimic biochemical communication, specifically within prototissues and protocellular materials. This mini-review aims to fill this gap. In particular, we have identified three categories, namely: 1) communication between distributed protocell populations; 2) communication within interconnected protocell networks; and 3) communication between protocells and living cells. We will critically discuss these three categories and attempt to draw important conclusions on their relative merits and future trajectories.

## Communication Between Distributed Protocell Populations

The molecular basis of multicellular life is thought to be linked to the ability of discrete, autonomous cells to signal their presence to one another; the evolution of cell signalling is thought to predate multicellularity (King et al., 2003). The development of protocells capable of chemical communication therefore represents a milestone in the development of fully functioning protocells and protocellular systems. The definition of inter-protocellular communication can be refined to the exchange of chemical signal between a “sender” and “receiver” protocell which causes an effect on the receiver. In the literature there have been a number of reports of communicating protocell populations which make use of a broad range of chemical systems to initiate and demonstrate their ability to send and receive chemical signals. The following examples have been further arranged into three subcategories: 1) simple response to diffusible signals; 2) computation and processing of chemical inputs to achieve greater complexity; and 3) the use of inter-protocellular communication to generate advanced biomimetic behaviours.

### Simple Response to Diffusible Signals

Communication between protocells should, at minimum, comprise the unidirectional transmission of information which results in a response in the receiving protocell. The first subdivision of communication between distributed protocells covers examples of protocells which respond in a “simple” fashion to a diffusible signal without significant processing of said signal.

**FIGURE 1:**
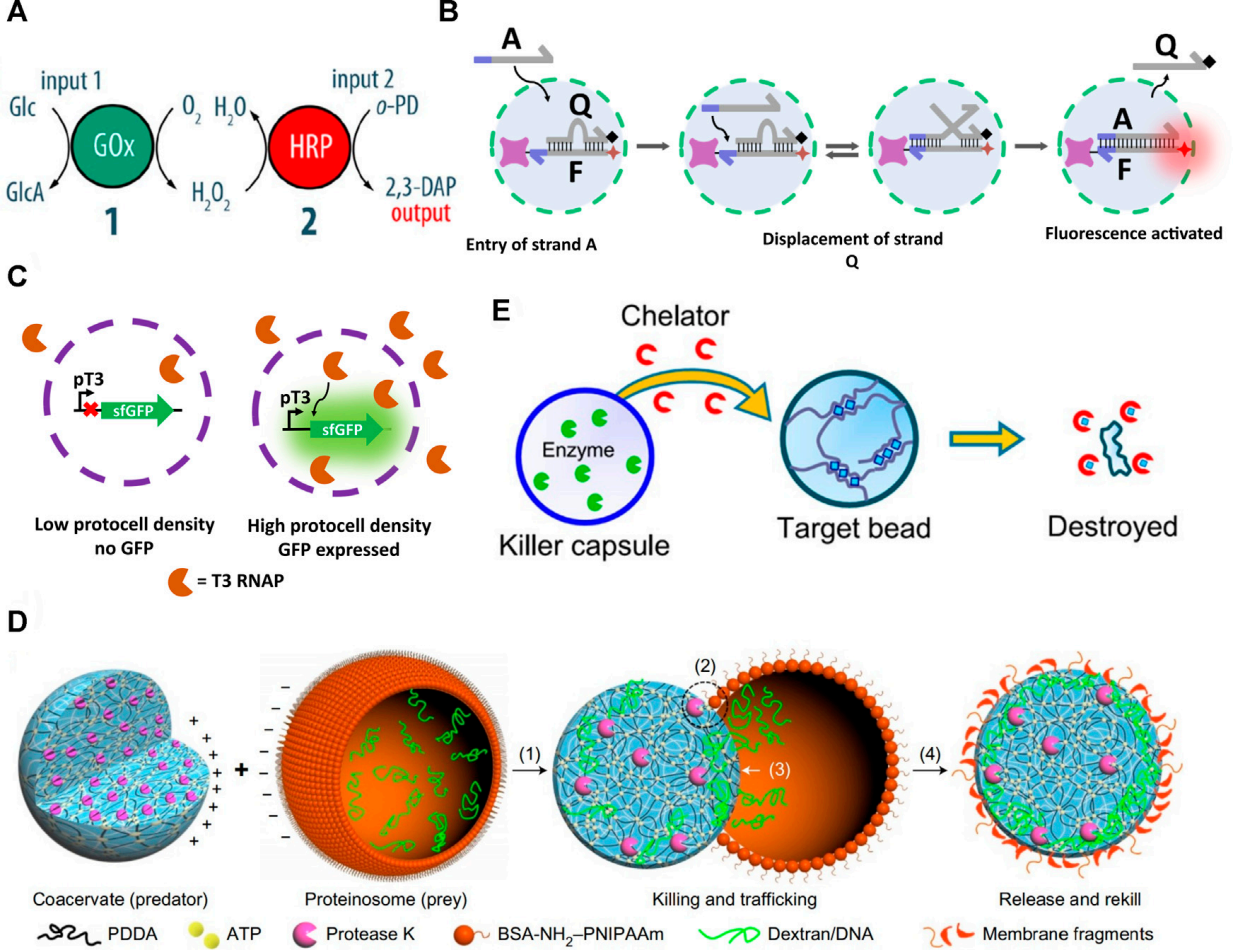
**(A)** Schematic describing a GOx/HRP enzyme cascade reaction between two populations of coacervate microdroplets. In this system the input signal is represented by glucose (Glc), which gets oxidised to cluconolactone (GlcA) by the GOx-containing coacervate microdroplet. This reaction produces the signalling molecule H_*2*_O_*2*_, which diffuses radially from the GOx containing coacervate microdroplet and is utilised by the HRP-loaded coacervate microdroplet to oxidise *o*-PD to 2,3-DAP and produce a fluorescent output signal (Tian et al., 2018). **(B)** Scheme describing the toehold DNA strand displacement communication mechanism between two proteinosomes. The input chemical signal represented by the single stranded DNA strand ‘A’ diffuses into a proteinosome containing a DNA gate complex and displaces strand Q which is functionalised with a fluorescence quenching moiety. The release of Q causes a fluorescence turn-on of the fluorophore which remains attached to the DNA gate complex inside the proteinosome (Joesaar et al., 2019). **(C)** Scheme describing quorum sensing in synthetic protocells. Polymer-based protocells contain plasmids coding for T3 RNA polymerase and for the green fluorescent reporter protein (sfGFP). Only at high densities of protocells in dispersion the local concentration of T3 RNA polymerase is high enough to trigger the transcription of sfGFP (Niederholtmeyer et al., 2018). **(D)** Scheme describing the predatory behaviour of coacervate microdroplets towards proteinosomes. The coacervate “predator” contains sequestered protease K. Electrostatic interactions bring positively-charged coacervate into contact with negatively-charged proteinosome “prey” (1). Protease K digests the protein-polymer material that composes the proteinosome’s membrane and so breaks apart the proteinosome (2). The “sponge-like” nature of the coacervate allows it to take up the membrane components of the proteinosome (3,4) (Qiao et al., 2017). **(E)** Scheme showing the reactivity of “killer” chitosan polymersomes. The killer polymersomes were loaded with GOx (green shapes) which converted glucose to gluconate “chelator” (red shapes). Gluconate diffused to the Cu^2+^-crosslinked “target” polymersomes and sequestered the copper ions (blue squares), causing the death of the target protocell (Arya et al., 2016). All figures adapted with permission.

The generation of a binary population of protocells made up of “senders” and “receivers” which communicate was demonstrated by L. Tian *et al.* in 2018, where coacervate microdroplets were used as a protocell model (Tian et al., 2018). Coacervates are self-assembled, membrane-free microdroplets comprising an aqueous phase rich in macromolecules held together by electrostatic interactions. Two populations of coacervate microdroplets were loaded with either horseradish peroxidase (HRP) or glucose oxidase (GOx) and were spatially trapped in periodic arrays using an acoustic standing wave pressure field. Glucose and *o*-phenylenediamine (*o*-PD), the substrates for the two enzymes, were co-diffused across the GOx-loaded protocell population first, which converted glucose into gluconolactone and generated the signalling molecule H_2_O_2_. H_2_O_2_ co-diffused with *o*-PD to the HRP-containing coacervates, which utilised H_2_O_2_ to oxidise *o*-PD to the fluorescent 2,3-diaminophenazine (2,3-DAP) (Figure 1A).

In contrast to the spatially organised coacervate model, T. Y. D Tang *et al.* reported the unidirectional transmission of chemical information between randomly dispersed typical giant unilamellar vesicles (GUVs) and a non-typical protein-polymer protocell (proteinosome) population (Tang et al., 2018). “Sender” GUVs used DNA transcription/translation machinery to produce a membrane pore upon addition of a small molecule transcriptional inducer that was able to diffuse across the GUV membrane. A pore-forming protein was then synthesised inside the GUV and allowed efflux of previously entrapped glucose, that was then able to freely diffuse until coming into contact with the receiver proteinosome population. The proteinosomes then transduced the chemical signal into a fluorescence output *via* an entrapped HRP/GOx enzyme cascade reaction which produced red fluorescent resorufin.

### Computation and Processing of Chemical Inputs

Living cells and organisms must be able to convert an external stimulus into a useful output. The more complex the cellular system and the higher the number of input signals, the greater the requirement for processing and interpretation of those inputs to allow for a greater range of possible responses by the living system. Following this general principle, researchers started to develop protocells capable of performing fundamental computational operations such as signal amplification and processing.

In living systems, proteins and other small molecules used for diffusible chemical signalling are often present at extremely low concentrations and their effect typically must be amplified by receiver cells to achieve signal transduction over large distances. A unidirectional signalling model between synthetic cells was developed by B. C. Buddingh and co-workers that emulated biological signal amplification (Buddingh’ et al., 2020). The group produced two populations of GUVs endowed with membrane pore proteins and either an enzyme for converting adenosine triphosphate (ATP) to adenosine monophosphate (AMP) (“sender protocell”) or an entrapped enzyme cascade initiated by glycogen phosphorylase (GP) and producing a fluorescent signal (NADH) as a final product (“receiver protocell”). The AMP signalling molecule diffused from the sender to the receiver protocells, converted the entrapped GP to its more active state through allosteric activation, and thus increased the activity of the entrapped enzyme cascade and NADH production. Crucially, 1 equivalent of AMP induced the production of 10 equivalents of NADH. Signal amplification is critical for the development of fully functioning networks of communicating protocells, as concentrations of diffusible signals become extremely low when transmitted over longer distances in solution.

Aside from signal amplification, the development of protocells capable of processing chemical signals is another important challenge in the field. T. F. A. de Greef and co-workers made a first important step to address this challenge: they made use of toehold DNA strand displacement (DSD) reactions to progressively convert chemical information passed through proteinosome populations (Zhang and Seelig, 2011; Huang et al., 2013; Joesaar et al., 2019). DNA information was held inside proteinosomes in “DNA gate complexes”. These were streptavidin protein centres functionalised with DNA duplexes where one of the two complementary ssDNA strands was directly attached to the protein *via* streptavidin/biotin interactions, whilst the second ssDNA molecule was bound through Watson-Crick base pairing to the first strand. An ssDNA strand with the correct toehold complementarity was perfused across the population of protocells and triggered the release of the previously bound ssDNA through a DSD reaction (Figure 1B). The use of DNA as a signalling molecule has clear benefits in terms of triggering a wide range of downstream effects, especially if combined with transcription/translation machinery or living cells. The high specificity of DNA could also be manipulated to generate highly complex signalling cascades and this work using DNA represents the first steps in the development of that complexity.

In more recent work by the same group, a similar proteinosome system was further enhanced by including a photoswitch in the gate complexes, adding a high degree of spatiotemporal control to the inter-protocellular communication. Instead of merely adding an initial ssDNA diffusible signal, a photolabile moiety – an *o-*nitrobenzyl group (*o*-NB)—was added to their DNA gate complexes which would trigger release of a ssDNA (Yang et al., 2020). This extra control was highly important because it allowed individual proteinosomes to be selectively induced to release signalling DNA strands to a population of “receiver” protocells. Furthermore, the authors were able to incorporate Boolean logic into their system using three distinct populations of proteinosomes, all loaded with a different gate complex: two were loaded with ssDNA signalling complexes and a third was loaded with a quenched duplex of DNA which required both ssDNA strands to initiate fluorescence. The inclusion of logic gates in these models is an important first step towards increasingly complex information processing within synthetic protocell communities. This more accurately emulates the vastly complex signal processing networks seen in all living cells and provides a basis for the development of future functional materials with information processing capacity.

### Emergence of Advanced Biomimetic Behaviours

Inter-protocellular signalling has been demonstrated in a variety of systems, some of which incorporate computation of the received signal. The next logical advance in bottom-up synthetic biology is the use of synthetic signalling systems to model advanced biomimetic behaviours – such as predation or quorum sensing.

A synthetic form of quorum sensing was developed using typical protocells synthesised *via* a microfluidic technique by H. Niederholtmeyer and co-workers who then used proteins as inter-protocellular signalling molecules (Niederholtmeyer et al., 2018). Quorum sensing in nature is found in bacteria to monitor their own population density through release and uptake of diffusible signals (Waters and Bassler, 2005; Albuquerque and Casadevall, 2012). These protocells constituted semipermeable microcapsules formed by polymerisation-induced phase separation and had ∼200 nm-sized pores through which macromolecules could diffuse (Kim et al., 2015). Using a system involving the highly promoter-specific T3 RNA polymerase (T3 RNAP) and a plasmid coding for a green fluorescent reporter protein, the authors caused the generation of fluorescent signals only when the density of individual protocell dispersions was sufficiently high. T3 RNAP was able to diffuse out of the protocells and transcribe the fluorescent protein gene under the control of the T3 promoter but, crucially, only when T3 RNAP was present at high enough concentration in the surrounding media, demonstrating rudimentary mimicking of quorum sensing (Figure 1C). Moreover, this work showed that communication between protocells is not limited to small diffusing molecules, but can also function effectively with macromolecules to achieve biomimetic behaviours.

A further complex biological behaviour, predation, was reported by Y. Qiao *et al.* who described a membrane-free coacervate protocell model “hunting” and “consuming” a proteinosome (Figure 1D). (Qiao et al., 2017) The coacervates used were loaded with a protease and, when mixed, were attracted to a population of negatively charged proteinosomes that the protease was able to digest. Due to the ability of coacervates to absorb molecules based on electrostatic interactions, the proteinosome membrane components and entrapped cargoes were subsequently sequestered within the coacervate. This work can be described as communication between cell mimics because the chemical information contained within the proteinosome was eventually taken up by the coacervate. Furthermore, this type of synthetic “communication” is the closest emulation of cell signalling through direct contact.

C. Arya and co-workers developed the predator/prey system with non-typical protocells made up of two different populations of polymersomes, one of which was made up of chitosan chains crosslinked both ionically and covalently, and the other of Cu^2+^-crosslinked alginate (Arya et al., 2016). The predatory chitosan polymersomes were loaded with GOx, and as glucose was added to a mixed population of both predator and prey polymersomes, gluconate was produced via GOx which competitively chelated the Cu^2+^ ions of nearby prey protocells, destroying them (Figure 1E). This, however, was highly location specific, occurring only between predator/prey couples that were appropriately close to each other.

The examples described here demonstrate the range of inter-protocellular signalling and rudimental cell-like behaviours that can be achieved with simple diffusible substrates. In living systems, cellular communication usually results in more complex changes such as gene expression, protein production or other significant biochemical outputs in the receiving cells. Different forms of output must therefore be developed in protocell engineering, not only to increase understanding of communication between living cells, but also to increase the range of systems available to applied synthetic biology. Communities of communicating protocells could have potential applications in diverse areas such as commercial biotechnology, pollutant removal or micro-scale sensing.

## Interconnected Protocell Networks

The next great advance in bottom-up synthetic biology is the construction of tissue-like structures from the controlled assembly of protocell units. Such assemblies of protocells can be held together using several different techniques and can display emergent properties.

**FIGURE 2:**
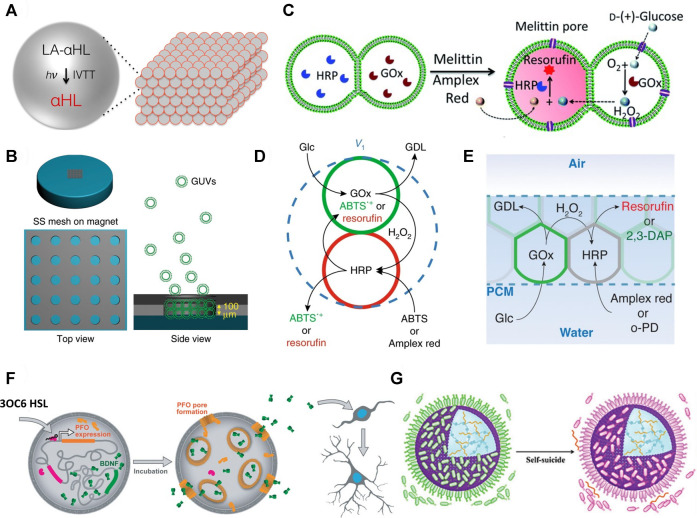
**(A)** Schematic of light activated communication within a 3D printed synthetic tissue. Light-activated α-hemolysin gene (LA-αHL) is expressed using *in vitro* transcription-translation system (IVTT). Once synthesised LA-αHL forms protein pores that allow for the exchange of substrates between protocells of synthetic tissues (Booth et al., 2016). **(B)** Formation of GUV-based prototissues using a magnetic field on a stainless steel (SS) grid (left). Magnetic GUVs (green round shapes) progressively assemble inside the grid to form prototissues guided by the magnetic field (right) (Li et al., 2020). **(C)** Scheme explaining the reactivity of prototissues comprising hemi-fused GOx- or HRP-loaded GUVs held together by acoustic pressure fields. Addition of melittin pore protein to this system allows entry of glucose and Amplex Red. Subsequently, glucose is oxidised to gluconolactone inside the GOx-loaded GUV and the signalling molecule H_2_O_2_ is produced. H_2_O_2_ diffuses into the other HRP-loaded GUV which catalyses oxidation of Amplex Red to red fluorescent resorufin (Wang et al., 2019). **(D)** Schematic showing diffusible communication between proteinosomes within a thermoresponsive prototissue spheroid. The input signal glucose (Glc) is converted to gluconolactone (GDL) by GOx inside the green proteinosomes. This reaction also releases the signalling molecule H_2_O_2_ which diffuses into the red proteinosomes containing HRP. HRP converts ABTS or Amplex red to fluorescent ABTS^•+^ or resorufin, respectively (Gobbo et al., 2018). **(E)** Schematic of chemical communication based on a GOx/HRP enzyme cascade reaction within a protocellular material. Details on the reactivity are reported in (d) (Galanti et al., 2021). **(F)** Mechanism of the chemical “translation” carried out by protocells for murine stem cells. The scheme shows a representation of the effects of released BDNF on murine stem cells. BDNF is constitutively expressed in the vesicle but cannot diffuse across its membrane. When 3OC6 HSL diffuses into the artificial vesicle, it initiates transcription and subsequent translation of PFO. PFO oligomerises and forms a pore in the membrane of the vesicle. This allows exit of BDNF from the vesicle. BDNF initiates development and branching of murine neural stem cells (Toparlak et al., 2020). **(G)** Scheme showing a proteinosome (purple sphere) inducing the death of multiple E. coli (green shapes = alive; pink shapes = dead). The positively charged proteinosome surface attracts the negatively charged bacteria. The death of the bacteria is induced through the contact with the ammonium salt present on the proteinosome’s membrane and through the pH-mediated release of chitosan oligosaccharides which are bactericides (Zhao et al., 2020). All figures adapted from original articles with permission.

An early fabrication of interconnected protocell networks was achieved by M. J. Booth and co-workers who 3D printed protocells as water-in-oil droplets, stabilised by fatty acids, and generated a synthetic model of neuronal electrical communication (Booth et al., 2016). The printed adjacent water droplets in oil formed an interfacial bilayer of fatty acids that mimicked a biological cell membrane. They were then able to incorporate light-activated gene transcription within specific protocell units that composed the tissue-like material. These protocells were capable of synthesising a pore-forming protein, which facilitated the exit of a fluorophore and exchange of ions under an applied electrical potential (Figure 2A).

Besides 3D printing, another method to control the spatial organization of protocells involves the application of a constant force field. For example, X. Han and co-workers synthesised diamagnetic GUVs and used a magnetic field across a stainless steel grid to assemble them into prototissues (Figure 2B) which were capable of inter-protocellular communication using a GOx/HRP enzyme cascade reaction and H_2_O_2_ as signalling molecule (Li et al., 2020). This magnetic method of assembling protocells was then used to produce prototissues capable of controlled release of nitric oxide (NO), which was exploited to trigger a vasodilatory response in murine bloods vessels. Similarly, Wang *et al.* used an acoustic trapping technique to assemble arrays of GUVs capable of communication *via* the addition of the protein pore melittin (Figure 2C). (Wang et al., 2019) Binary populations of GUVs could be co-localised and their membranes partially fused upon addition of Ca^2+^. The system was utilised further to induce death in cancer cells via H_2_O_2_ release or bacterial gene expression through controlled release of the signalling molecule IPTG. Whilst these represent excellent examples of networks of interconnected protocells capable of communication and displaying emergent properties, the reliance on a constant magnetic or acoustic pressure field to maintain the 3D architecture of the tissue-like structure could limit the future utility of such assemblies.

In contrast to the 3D printing and external force field techniques outlined above, P. Gobbo and co-workers developed a method for the programmed assembly of binary protocell communities into tissue-like materials based on the formation of covalent protocell-protocell adhesions. For this, they used proteinosomes as the protocell model and functionalised their membranes with either azide or strained alkyne groups for an interfacial strain-promoted alkyne-azide cycloaddition (I-SPAAC) reaction (Gobbo et al., 2013). They were then able to assemble these chemically reactive protocells into small prototissue spheroids using a recursive Pickering emulsion technique.

The thermoresponsive properties of the individual proteinosome units were then exploited collectively to generate prototissue spheroids with emergent contractile properties that could be enzymatically modulated or exploited for mechanochemical transduction (Figure 2D). Building on this work (Gobbo et al., 2018), K. Ramsay and co-workers showed that microfluidics can be exploited for the generation of bespoke prototissue spheroids with high control over their size, composition and with unique Janus configurations (Ramsay et al., 2021). Most importantly, by controlling the number and type of the protocells that composed the spheroids, they could control both the amplitude of the biomaterial’s thermally induced contractions and its collective endogenous biochemical reactivity. Finally, our group recently reported a novel technique, termed the “floating mould technique”, for the controlled assembly of the first “protocellular materials” (PCMs) with complex 3D architectures. PCMs are centimetre sized assemblies of covalently ligated azide- and strained alkyne-functionalised protocells that are robust, free standing, stable in water, and capable of chemical communication (Figure 2E). (Galanti et al., 2021) Most importantly, PCMs can be easily manipulated by hand making them ideal candidates for a variety of different applications from materials science to regenerative medicine.

The combination of protocells into interlinked networks is clearly a step forward in bottom-up synthetic biology, and from a more practical perspective, it has evident benefits in terms of mechanical strength and appears to result in emergent collective protocell behaviours. Thus far, the scope of potential communication pathways is highly limited, relying heavily on the HRP/GOx cascade reaction and on H_2_O_2_ as the signalling molecule. Therefore, expansion of communication capabilities will be required in order to produce novel bio-inspired materials with more advanced bio-mimetic functionalities. Despite this current limitation, the ability to construct functional macroscale materials from the controlled assembly of microscopic protocell units has the potential to revolutionise the field of synthetic biology and materials science; the incorporation of multi-compartmentalisation into materials allows for the development of modularity, which in turn will lead to unprecedented functional complexity.

## Communication Between Protocells and Living Cells

Of the future applications of protocell technology, drug delivery and regenerative medicine must surely be one of the most exciting. Therefore, the ability of protocells to interact and respond to biological cells and tissues is crucial to realising these applications. To date, this area of research is limited – there are few examples reported in the literature, mostly describing the use of protocells as “translators” of chemical information for living cells. Moreover, in recent years, the standard approach to modifying the behaviour of consortia of cells has been through direct manipulation of genetic material; protocells could provide a viable alternative method for the control or management of cells without the need for difficult and expensive genome engineering in the organism of interest.

An example of this was described by R. Lentini and co-workers who “translated” chemical information into “readable” form for a population of *E. coli* (Lentini et al., 2014). The protocells used were artificial vesicles, synthesized from phospholipids and containing DNA, transcription-translation machinery and isopropyl *ß*-_D_-1-thiogalactopyranoside (IPTG)—a signalling molecule that *E. coli* are naturally responsive to. The DNA sequence coded for a known riboswitch and a fluorescent pore-forming protein, resulting in the production of fluorescent protein only in the presence of theophylline (Lynch and Gallivan, 2009). When theophylline was introduced to the mixed population of protocells and bacteria, pores formed in the protocells’ lipid membranes, allowing efflux of the pre-loaded IPTG. This signalling molecule diffused to *E. coli* and initiated a response, therefore the IPTG-containing protocells *translated* a chemical input for the bacteria. A related example was reported by G. Rampioni *et al.* who triggered a bioluminescent response in *P. aeruginosa* using synthetic cell-controlled release of a homoserine lactone signal (Rampioni et al., 2018).

Similarly, O. D. Toparlak and co-workers showed that neuronal development in murine neural stem cells can be initiated by the synthesis and release of a biological neurotrophic factor from protocells (Toparlak et al., 2020). In this work, a synthetic phospholipid vesicle was loaded with transcription-translation machinery as well as a genetic sequence that coded for a neurotrophic factor, a membrane pore-forming protein, and a transcriptional repressor. In the presence of a small molecule (*N*-3-oxohexanoyl homoserine lactone, 3OC6 HSL) the pore-forming protein would be expressed inside the vesicles and allowed the diffusion of the neurotrophic factor into the surrounding media (Figure 2F). The protocells were incubated with neural stem cells and induced differentiation following the input of 3OC6 HSL. The protocell system also had an effect on HEK293 cells under physiological conditions, with implications in the field of drug delivery – HEK293 cells are one of the most intensively researched human cell lines in existence, with particular significance in cancer biology (Stepanenko and Dmitrenko, 2015).

Although the “translation” of chemical information into a readable form for living cell populations has medical applications, the construction of “predatory” protocells capable of “hunting” living cells would also have potential in medicine, for example in the treatment of cancers and parasitic or bacterial infections. A rudimentary mimicking of predatory behaviour has been demonstrated by C. Zhao *et al.* who developed proteinosomes that caused the death of up to hundreds of *E. coli* (Zhao et al., 2020). The proteinosome membranes attracted individual bacteria through electrostatic interactions. Once adsorbed onto the proteinosome surface, the metabolism of the *E. coli* generated a localised acidic environment, triggering the breakdown of a hydrogel inside the proteinosome. Sequestered in the hydrogel were chitosan oligosaccharides grafted to _l_-arginine which, on release, caused widespread bacterial cell death, not only in the immediate vicinity of the proteinosome, but also of unadsorbed *E. coli* (Figure 2G).

Although a few examples of communication between living and artificial cells are reported in the literature, this area of protocellular communication is understandably limited. The potential applications, for medicine in particular, could be profound if more research was conducted in this area. In order to initiate communication with living cells, the strategy of two out of the three examples described above simply causes the release of a chemical effector from a vesicle through a protein pore. This method seems overly complex, as to achieve protein pore formation DNA transcription-translation machinery must be employed within the protocell which is both costly and restrictive in terms of environmental conditions available. The same effect could surely be achieved with a simpler method of controlling egress of signalling molecules from protocells. Furthermore, the communication demonstrated has only dealt with protocell/cell communication, and protocells have yet to combined with larger biological materials–*e.g.* tissues or multicellular organisms. This emerging subfield certainly has exciting potential and provides many opportunities for further research.

## Conclusion

The development of multi-protocellular systems (either dispersed or interconnected) capable of sending and receiving chemical signals is essential to spearhead the development of important real-world applications for protocells and tissue-like materials (Correia Carreira et al., 2020). The triggering of individual or collective cell-like behaviours or of communication with living cells and tissues in a controlled manner has vast potential in applied science and technology. For example, we can envision the possibility of delivering targeted therapies either *in vitro* or *in vivo*; developing advanced scaffolds for tissue engineering capable of influencing cell growth, proliferation and differentiation; and manipulating organisms’ metabolism using “translation” systems while avoiding direct interference in genomic material.

This type of research will not only lead to important applications in biotechnology but also has the potential to revolutionise the field of materials chemistry. Indeed, the methodologies to synthesise and characterise bio-inspired networks of communicating protocells that will derive from the evolution of this research will also have a significant impact on emerging areas of materials science such as the engineering of active matter (Needleman and Dogic, 2017; Rubio-Sánchez et al., 2021), the fabrication of intelligent materials (Kaspar et al., 2021), the transduction of light to chemical energy (Altamura et al., 2017; Berhanu et al., 2019; Biner et al., 2020; Altamura et al., 2021b) and the development of advanced materials capable of unconventional computations (Adamala et al., 2017; Lyu et al., 2018; Dubuc et al., 2019; Dupin and Simmel, 2019; Bartoli et al., 2020; Green et al., 2021). Another important area of application is soft robotics. The possibility of developing tissue-like materials that can transduce a chemical stimulus into a programmed mechanical movement will allow for the development of advanced soft-bots or soft robotic components that can react to external stimuli or even be fuelled by specific compounds available in the environment (Downs et al., 2020; Gobbo, 2020).

It is evident how the emerging area of bioinspired networks of communicating protocells provides a plethora of avenues for future research directions with many research fields that could benefit from it. However in the forthcoming years, research efforts must be put into designing and synthesising more advanced communication pathways, especially between non-living and living matter.
